# Supplementary material for: Multi-task machine learning improves multi-seasonal prediction of the Indian Ocean Dipole
Source: Nat Commun. 2022 Dec 12;13:7681. doi: 10.1038/s41467-022-35412-0 (PMC9744903; doi:10.1038/s41467-022-35412-0)
Supplement: Supplementary file 1 — Supplementary Information [file 41467_2022_35412_MOESM1_ESM.pdf]

Multi-task Machine Learning Improves Multi-Seasonal Prediction of the Indian Ocean Dipole

Supplementary Information

Fenghua Ling[1], Jing-Jia Luo[1*], Yue Li[1], Tao Tang[1], Lei Bai[2], Wanli Ouyang[2,3], and Toshio Yamagata[1,4]

[1]Institute for Climate and Application Research (ICAR)/CIC-FEMD/KLME/ILCEC, Nanjing University of Information Science and Technology, Nanjing, China
[2]Shanghai AI Laboratory, Shanghai, China
[3]School of Electrical and Information Engineering, The University of Sydney, Sydney, NSW, Australia
[4]Application Laboratory, Japan Agency for Marine-Earth Science and Technology, Yokohama, Japan

*Corresponding to jjluo@nuist.edu.cn; jingjia_luo@hotmail.com

Including:

Supplementary Table 1.

Supplementary Table 2.

Supplementary Fig.1.

Supplementary Fig. 2.

Supplementary Fig. 3.

Supplementary Fig. 4.

Supplementary Fig. 5.

Supplementary Fig. 6.

Supplementary Fig. 7.

Supplementary Fig. 8.

Supplementary Fig. 9.

Supplementary Fig. 10.

Supplementary Fig. 11.

Supplementary Fig. 12.

Supplementary Table 1. Centennial historical simulations from Coupled Model Intercomparison Project phase 5 and 6 (CMIP5/6) models that are used to train the multi-task learning model

CMIP5			
Model name	Institution	Simulation period	Number of Ensemble members
BCC-CSM1.1-m	Beijing Climate Center, China Meteorological Administration	1860-2003	1
CanESM2	Canadian Centre for Climate Modelling and Analysis	1860-2003	5
CMCC-CM	Centro Euro-Mediterraneo per I Cambiamenti Climatici	1860-2003	1
CMCC-CMS		1860-2003	1
CNRM-CM5	Centre National de Recherches Meteorologiques /Centre Europeen Recherche et Formation Avancee en Calcul Scientifique	1860-2003	5
GFDL-ESM2G	NOAA Geophysical Fluid Dynamics Laboratory	1860-2003	1
GISS-E2-H	NASA Goddard Institute for Space Studies	1860-2003	5
HadGEM2-AO	National Institute of Meteorological Research/Korea Meteorological Administration	1860-2003	1
HadGEM3	Met Office Hadley Centre (additional HadGEM2-ES realizations contributed by Instituto Nacional de Pesquisas Espaciais)	1860-2003	1
HadGEM2-CC		1860-2003	1
HadGEM2-ES		1860-2003	4
IPSL-CM5A-MR	Institut Pierre-Simon Laplace	1860-2003	1
MIROC5	Atmosphere and Ocean Research Institute (The University of Tokyo), National Institute for Environmental Studies, and Japan Agency for Marine-Earth Science and Technology	1860-2003	1
MPI-ESM-LR	Max-Planck-Institut fur Meteorologie (Max Planck Institute for Meteorology)	1860-2003	3
MRI-CGCM3	Meteorological Research Institute	1860-2003	1
NORESM1-M	Norwegian Climate Centre	1860-2003	1

NORESM1-ME		1860-2003	1
CMIP6			
Model name	Institution	Simulation period	Number of Ensemble members
BCC-CSM2-MR	Beijing Climate Center, China Meteorological Administration	1860-2009	1
BCC-ESM1		1860-2009	1
CanESM5	Canadian Centre for Climate Modelling and Analysis	1860-2009	1
EC-Earth3-Veg	European Centre for Medium-Range Weather Forecasts	1860-2009	1
EC-Earth3		1860-2009	1
GISS-E2-1-G	NASA Goddard Institute for Space Studies, USA	1860-2009	1
GISS-E2-1-H		1860-2009	1
IPSL-CM6A-LR	Institut Pierre Simon Laplace, France	1860-2009	1
MIROC6	Japan Agency for Marine-Earth Science and Technology/Atmosphere and Ocean Research Institute, Japan/ National Institute for Environmental Studies, Japan/RIKEN Center for Computational Science, Japan	1860-2009	1
MPI-ESM-1-2-HAM	Max Planck Institute for Meteorology, Germany	1860-2009	1
MPI-ESM-1-2-HR		1860-2009	1
MPI-ESM-1-2-LR		1860-2009	1
MRI-ESM2-0	Meteorological Research Institute, Japan	1860-2009	1
NorESM2-LM	Norwegian Climate Centre, Norway	1860-2009	1
NorESM2-MM		1860-2009	1

Supplementary Table 2. List of the datasets that the multi-task learning model (MTL-NET) used

	Data	Period
Training dataset	CMIP5/6 historical simulations	1860-2009
Transfer dataset	Reanalysis (SODA, NOAA 20CR V3)	1871-1973
Test dataset	Observations (GODAS, NCEP Reanalysis II)	1983-2019

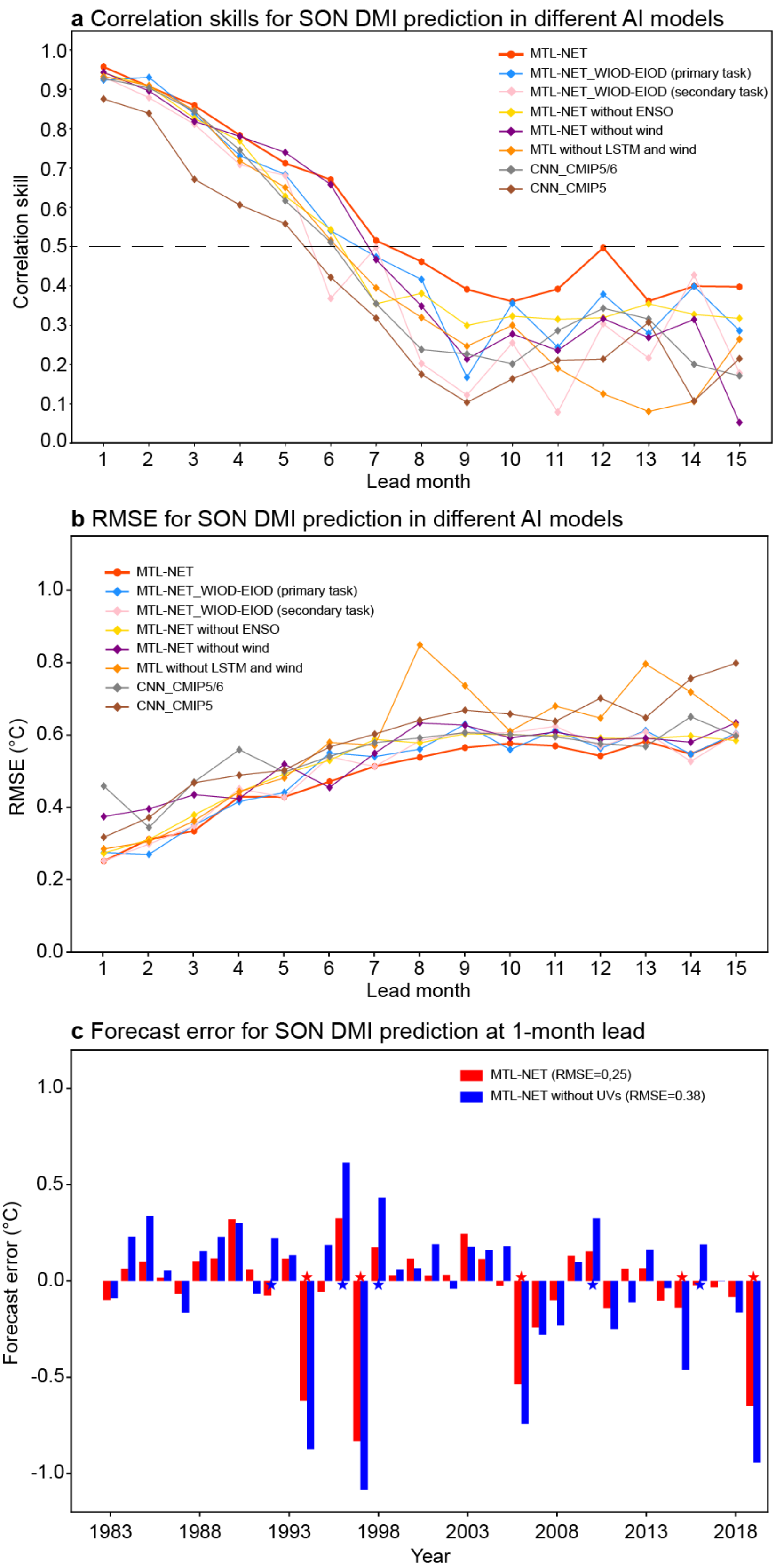

Supplementary Fig. 1. Indian Ocean Dipole prediction skills based on different machine learning models. (a) Predictive skill of the Sep-Oct-Nov (SON) dipole mode index (DMI) as a function of forecast lead month in different models. These models are:

the multi-task learning model (MTL-NET, red line; i.e., the best model adopted in this study); the MTL-NETs which set the sea surface temperature anomaly of western pole of the Indian Ocean (WIOD) and eastern pole of the Indian Ocean (EIOD) as primary task (i.e., WIOD minus EIOD, blue line) and the secondary tasks (pink line), respectively; the MTL-NET without ENSO task (yellow line); the MTL-NET without surface wind (Us/Vs) predictors (purple line); the MTL-NET without either Us/Vs predictors or the LSTM block (orange line); the CNN model using CMIP5 dataset only (brown line) and the CNN model using both CMIP5 and 6 datasets (gray line) for training. The prediction skill is validated for the period of 1983-2019. (b) As in (a), but for the root mean square error (RMSE). (c) Forecast errors (i.e., predictions minus observations) of the SON DMI based on the MTL-NET (red bars) and the MTL-NET without Us/Vs predictors (blue) at one month lead. The stars represent the years of extreme IOD events, with the red (blue) star representing a positive (negative) IOD. All results are based on 10-member ensemble mean.

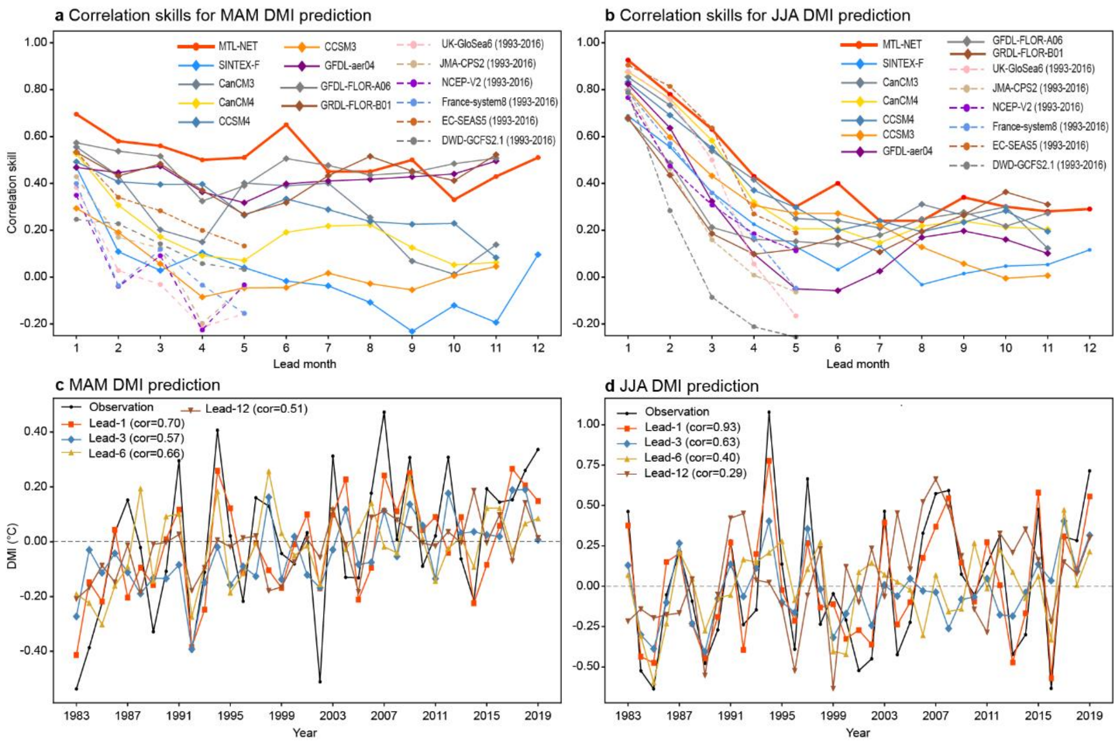

Supplementary Fig. 2. Correlation skills in predicting the Dipole Mode Index (DMI) in Mar-Apr-May (MAM) and Jun-Jul-Aug (JJA). (a) Predictive skill of the MAM DMI as a function of the forecast lead month in the multi-task learning model (MTL-NET, red line), SINTEX-F dynamical forecast system (blue lines), six operational forecast models which hindcast period is limited to the period after 1993 (dashed lines) and seven dynamical forecast systems of the North American Multi-Model Ensemble (NMME) project (the other coloured lines). The validation period is from 1983 to 2019. (b) As in (a), but for the predictions of JJA DMI. (c, d) The DMI in MAM and JJA based on the observations (black line) and 10-member ensemble mean predictions of MTL-NET at lead time of 1, 3, 6 and 12 months (red, blue, yellow and brown lines), respectively.

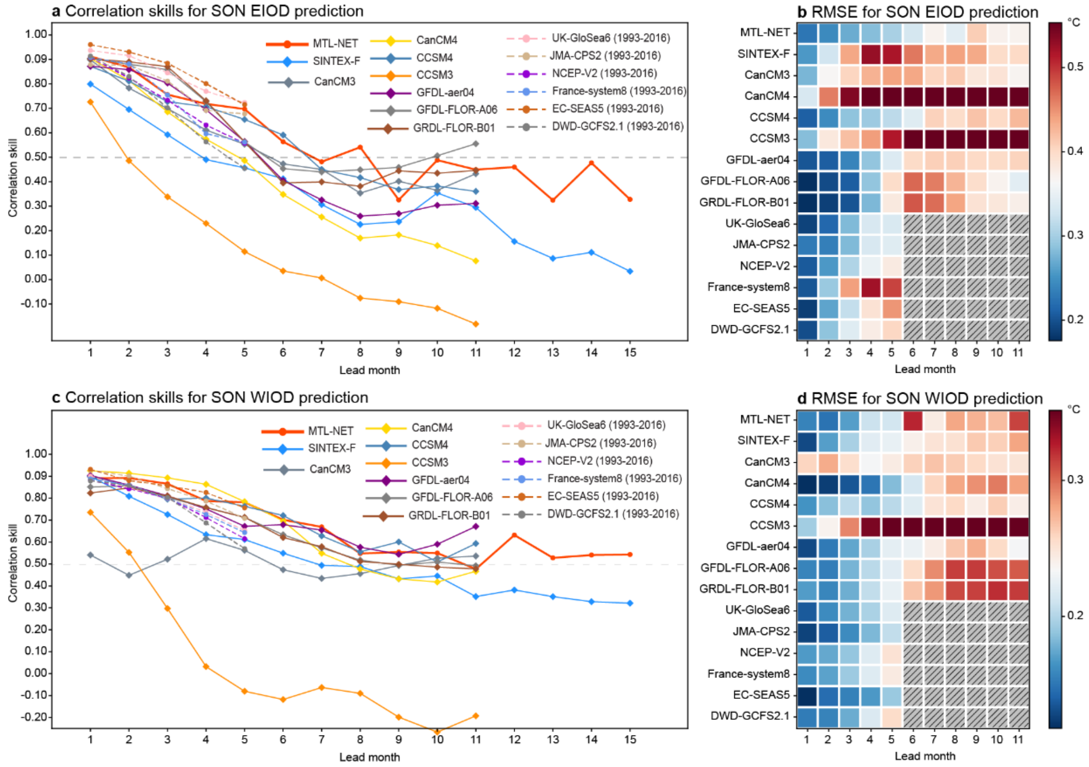

Supplementary Fig. 3. Correlation skills in predicting the sea surface temperature anomaly of eastern pole of the Indian Ocean (EIOD) and western pole of the Indian Ocean (WIOD) in Sep-Oct-Nov (SON). (a, c) As in Supplementary Fig. 2a, but for the predictive skill of the SON EIOD and WIOD indices, respectively. (b, d) RMSE of the predictions of the SON EIOD and WIOD indices based on the multi-task learning model (MTL-NET) and individual dynamical model forecast systems.

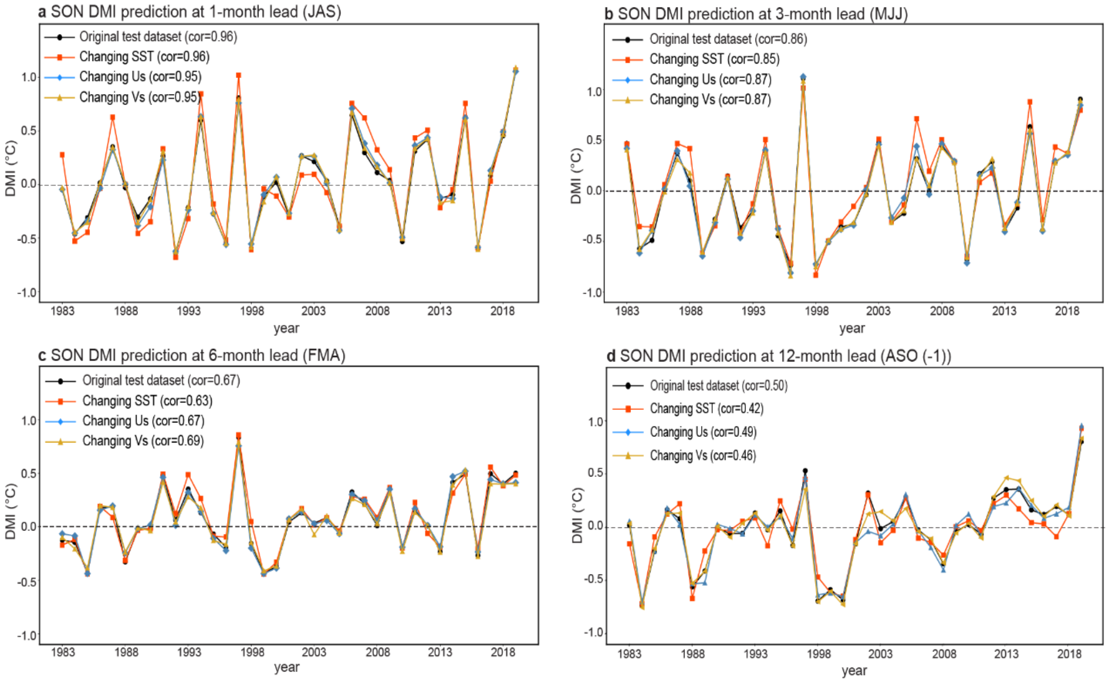

Supplementary Fig. 4 The robustness of the multi-task learning model (MTL-NET) predictions. (a-d) The Sep-Oct-Nov Dipole Mode Index (SON DMI) predicted at 1-, 3-, 6-, and 12-month lead based on the original data (original test dataset, black lines), different sea surface temperature (SST), surface zonal wind (Us), and surface meridional wind (Vs) data (red, blue, yellow lines), respectively (see “Sensitivity experiments” in Methods). All results are based on 10-member ensemble mean.

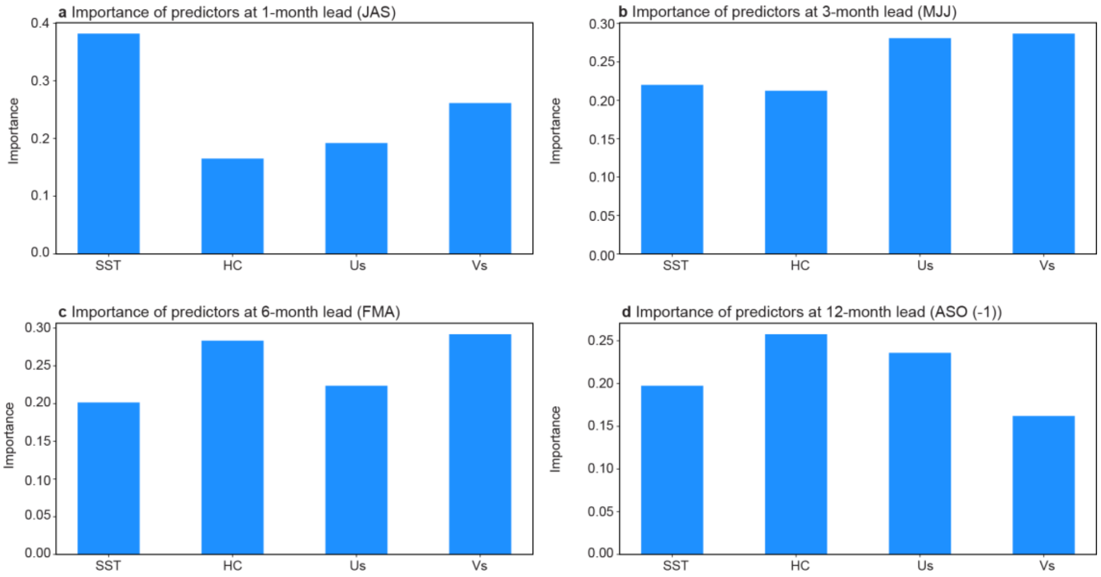

Supplementary Fig. 5. The importance of the predictors estimated by the multi-task learning model (MTL-NET) at different lead times. The results are produced by the channel attention mechanism for the Dipole Mode Index (DMI) predictions in Sep-Oct-Nov initiated from (a) Jul-Aug-Sep (JAS), (b) May-Jun-Jul (MJJ), (c) Feb-Mar-Apr (FMA), and (d) Aug-Sep-Oct (ASO (-1)) in previous year, respectively.

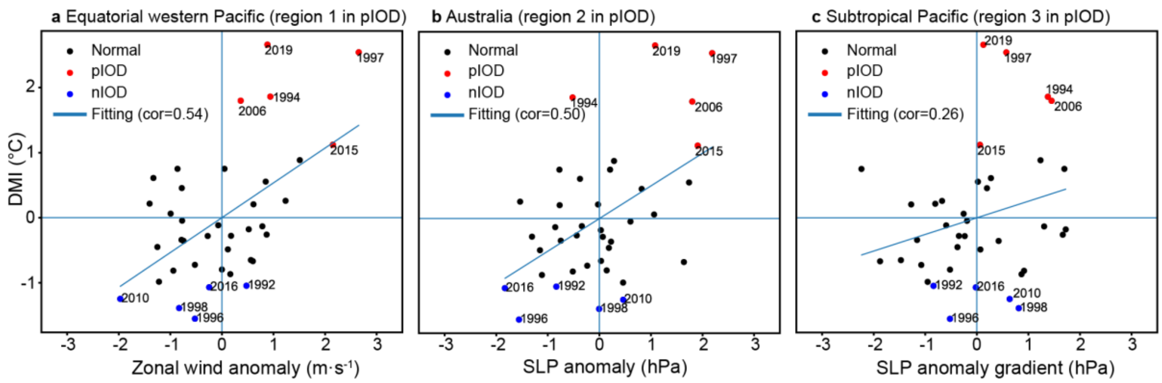

Supplementary Fig. 6. The relations between the predictors at 3-month lead and the five strong positive Indian Ocean Dipole (pIOD) events over the past four decades. (a) The scatter plot of May-Jun-Jul zonal wind anomaly in the equatorial western Pacific (i.e., region 1 in Fig. 3a, 135°E-200°E,10°S-10°N) against the Dipole Mode Index (DMI) in Sep-Oct-Nov. The pIOD, negative IOD (nIOD), and neutral years are indicated by red, blue, and black points, respectively. (b, c) As in (a), but for the results based on MJJ sea-level pressure (SLP) anomaly in Australia (i.e., region 2 in Fig. 3a, 110°E-180°E, 45°S-10°S) and SLP anomaly gradient between subtropical northwestern Pacific (region 3 in Fig. 3a, 150°E-180°E, 20°N-40°N) and tropical northwestern Pacific (150°E-180°E, 10°N-20°N).

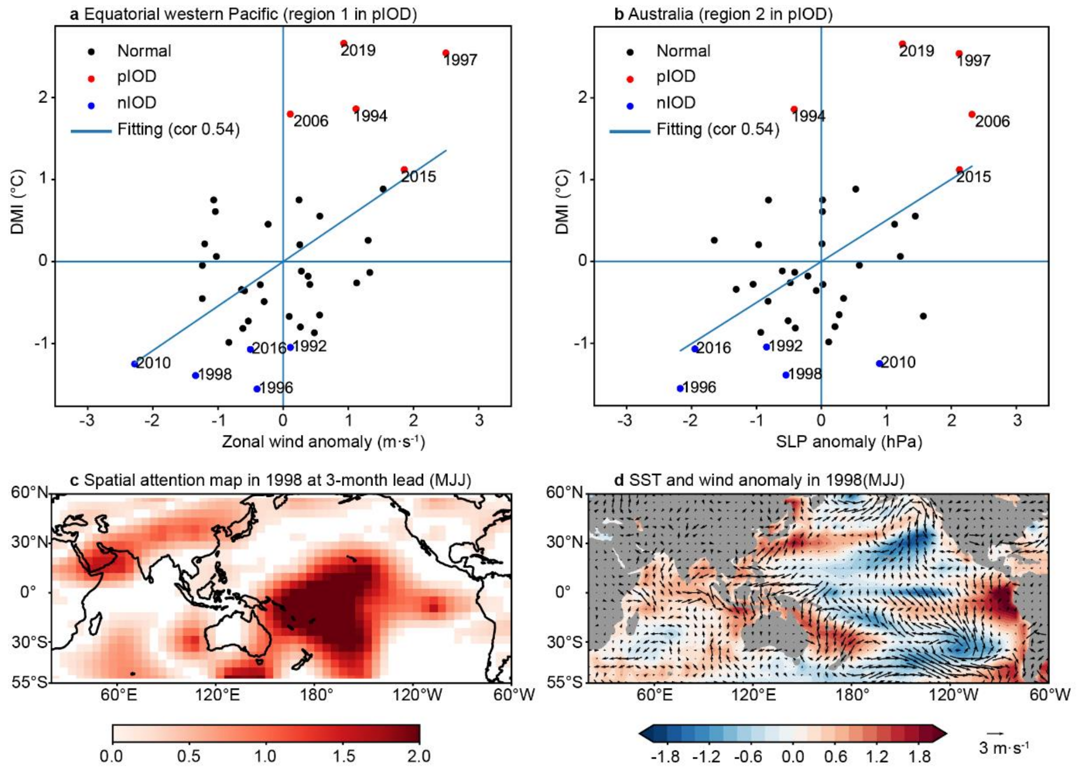

Supplementary Fig. 7. The relations between the predictors at 3-month lead and the five strong negative Indian Ocean Dipole events (nIOD) over the past four decades. (a) The scatter plot of May-Jun-Jul (MJJ) zonal wind anomaly in the equatorial western Pacific (i.e., region 1 in Fig. 3b, 115°E-190°E,10°S-15°N) against the Dipole Mode Index (DMI) in Sep-Oct-Nov. The positive IOD events (pIOD), nIOD, and normal years are indicated by red, blue, and black points, respectively. (b) As in (a), but for the results based on MJJ sea-level pressure (SLP) anomaly in Australia (i.e., region 2 in Fig. 3b, 100°E-165°E, 45°S-15°S). (c) The spatial attention map of the multi-task learning model (MTL-NET) forecasts of 1998 nIOD event initialized from MJJ (namely 3-month lead). Only the values significant at the 90% confidence level based on 10 ensemble members are displayed. (d) The observed sea surface temperature (SST) anomalies (shaded) and surface wind (vector) anomalies in MJJ 1998.

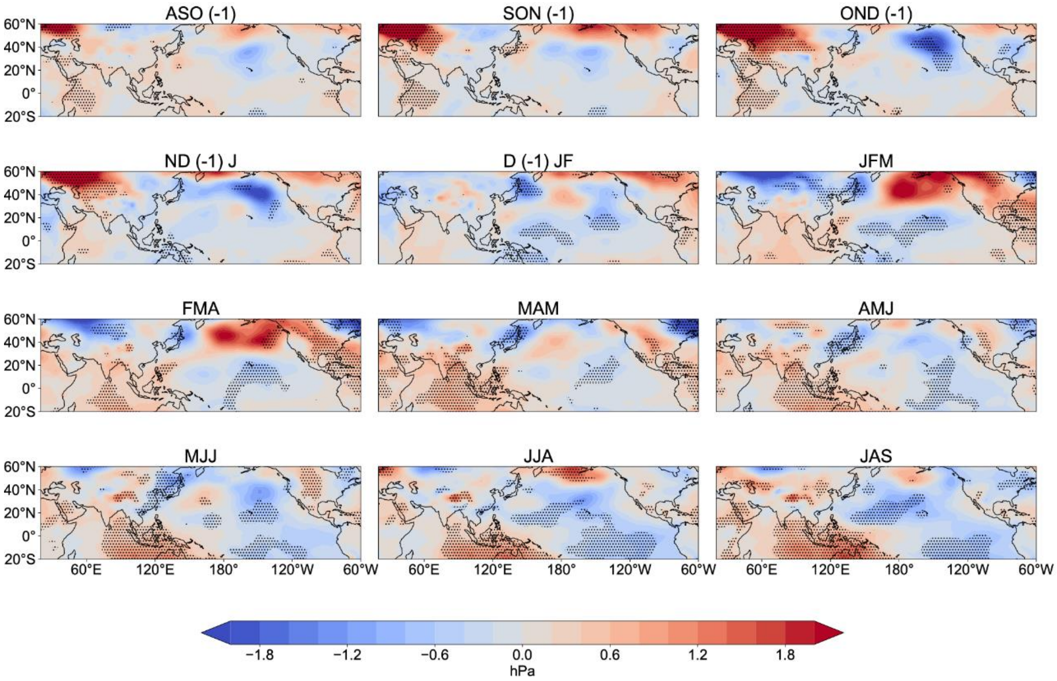

Supplementary Fig. 8. The evolution of sea-level pressure (SLP) anomalies of the five strong positive Indian Ocean Dipole events (pIOD) over the past four decades (i.e., 1994, 1997, 2006, 2015, and 2019). The composite maps of 3-month running mean SLP anomalies prior to the pIOD peak phase from Aug-Sep-Oct of preceding year (ASO (-1)) to Jun-Aug-Sep (JAS) of concurrent year. Stippling denotes the areas where the anomalies are significant at the 10% level according to the Student's t-test.

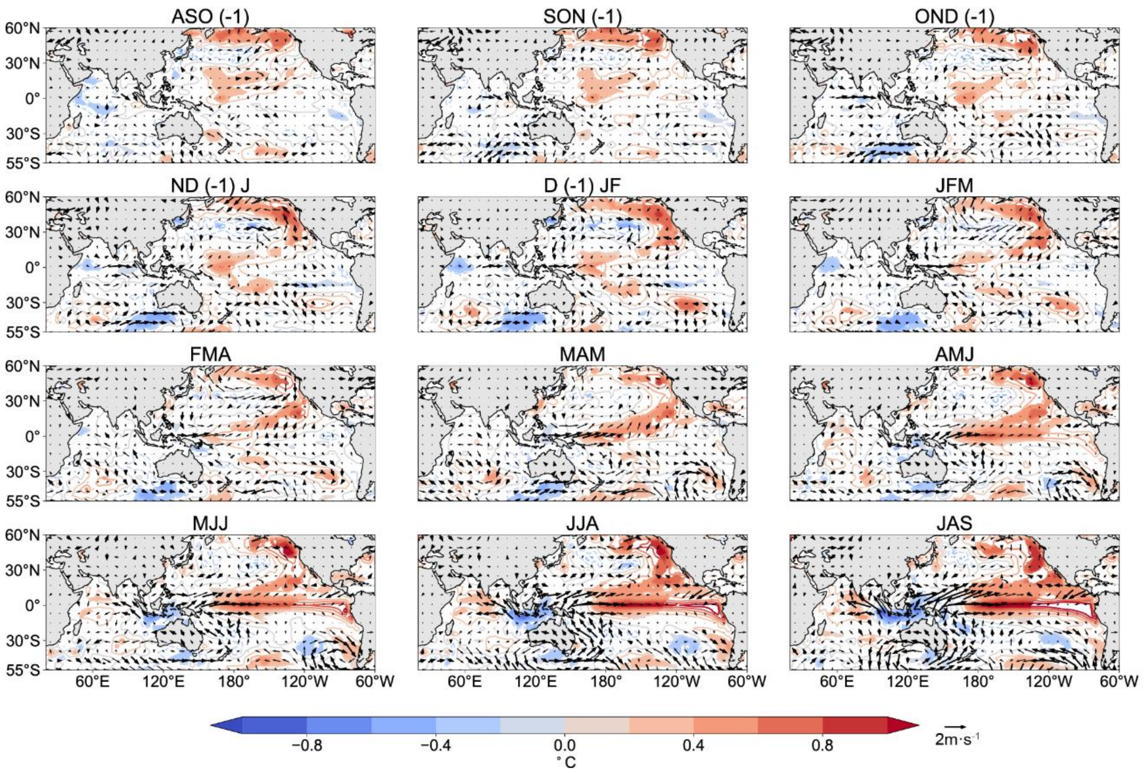

Supplementary Fig. 9. The evolution of sea surface temperature (SST) and wind anomalies associated with the five strong positive Indian Ocean Dipole events (pIOD). As in Supplementary Fig. 8, but for the 3-month running mean anomalies of SST (shading and contour) and surface winds (vector). The vectors in bold and shading denote the areas where the anomalies are significant at 10% level according to the Student's t-test.

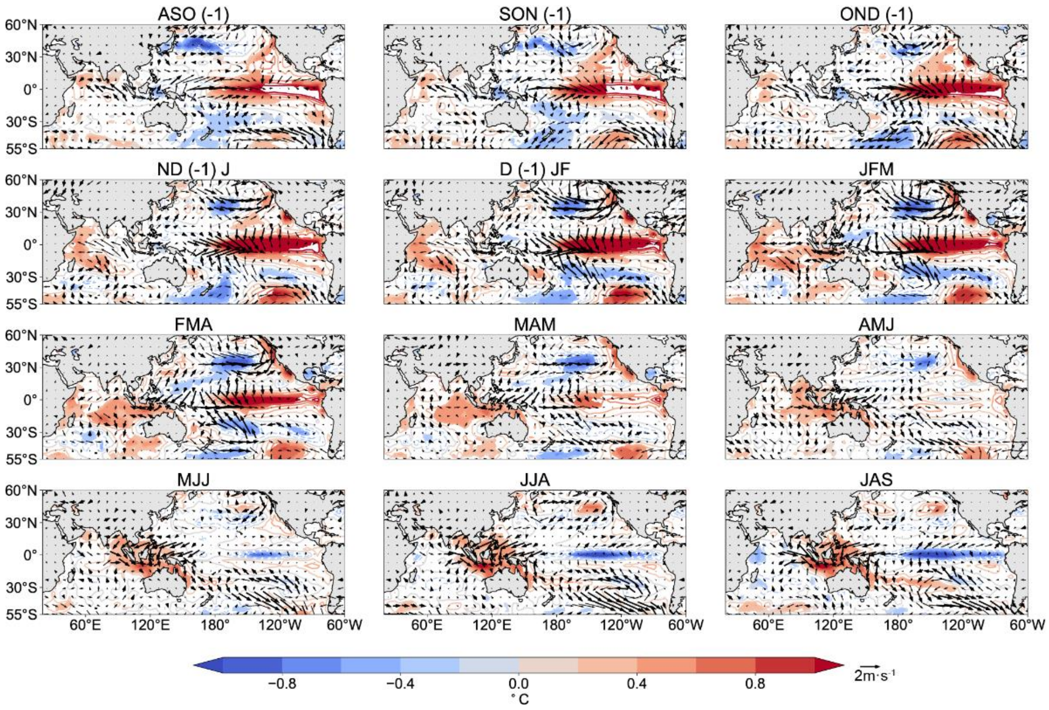

Supplementary Fig. 10. The evolution of sea surface temperature (SST) and wind anomalies associated with the five strong negative Indian Ocean Dipole events (nIOD). As in Supplementary Fig. 9, but for the results for the five nIOD events.

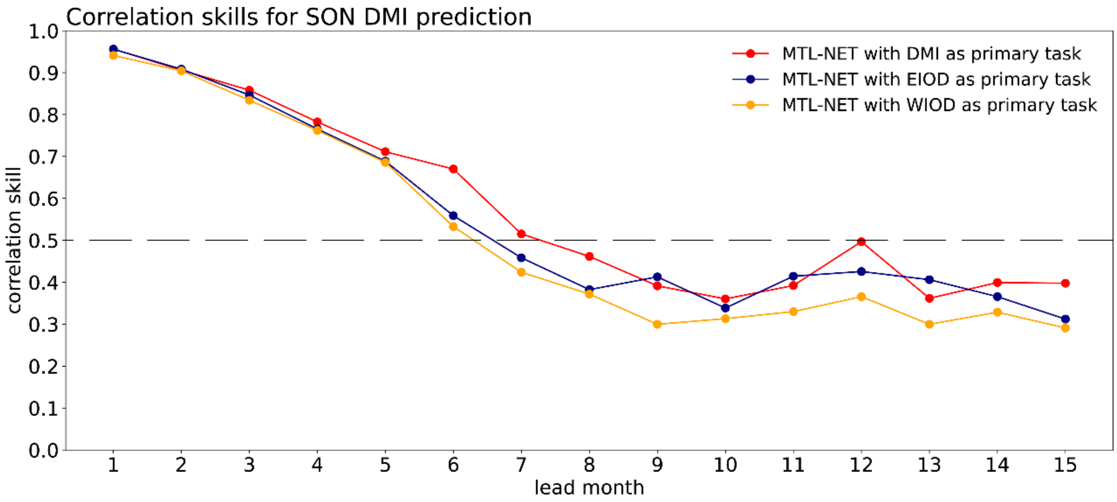

Supplementary Fig 11. Prediction skills for the Dipole mode Index (DMI in Sep-Oct-Nov based on multi-task learning model (MTL-NET) with different predictand as primary task. Predictive skill of the SON DMI as a function of the forecast lead month in the MTL-NET with DMI being set as the primary task (red line), MTL-NET with the sea surface temperature anomaly of eastern pole of the Indian Ocean (EIOD) as the primary task (blue line), and MTL-NET with the SST anomaly of western pole of the Indian Ocean (WIOD) as the primary task (orange line). The validation period is from 1983 to 2019.

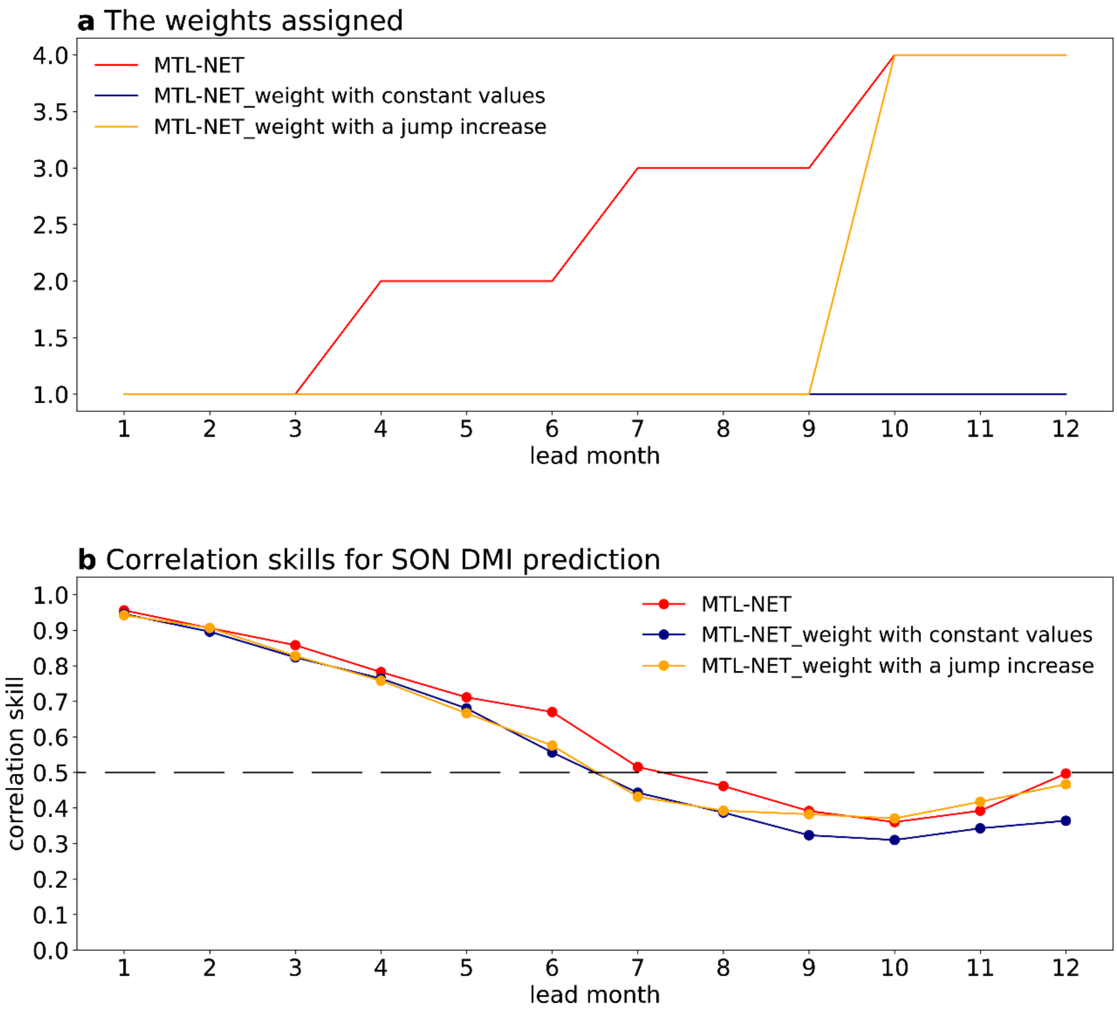

Supplementary Fig 12. Prediction skills based on different weights set for the El Niño/Southern Oscillation (ENSO) task in the loss function. (a) The different weights set for the ENSO task in the loss function. (b) The correlation skill of the dipole mode index (DMI) prediction in Sep-Oct-Nov (SON) based on the different ways of setting the weights in the loss function. The validation period is from 1983 to 2019.
